# Comparison of patient-reported adverse drug reactions with pharmacovigilance records in gastrointestinal disorders

**DOI:** 10.6026/973206300222035

**Published:** 2026-04-30

**Authors:** Adithi Umesh Basappa, Nandita Tallam, Riyaz Siddiqui, Dhivya Mohan, Manish Ramesh Bhise, Aisha Mohammed Kutty

**Affiliations:** 1 Department of Emergency Medicine, ETCM Hospital, Karnataka, India; 2 Department of Anatomy, Mediciti institute of medical sciences, Telangana, India; 3 Department of Pharmacology, NKP Salve Institute of Medical sciences & RC and LMH, Nagpur, India; 4 Department of Medicine, Aarupadai Veedu Medical College and Hospital, Tamil Nadu, India; 5 Department of Pharmaceutics, SGSPS, Institute of Pharmacy, Akola, Affiliated to Sant Gadge Baba Amravati University, India; 6 Department of Oncology and Haematology, Portsmouth University Hospital NHS trust, Wessex, United Kingdom, India

**Keywords:** Adverse drug reaction (ADR), patient-reported outcome, pharmacovigilance, gastrointestinal disorder, drug safety, perception survey, self-reported experience, medication side effect, signal detection and questionnaire-based study

## Abstract

Adverse drug reactions (ADRs) are frequently under-recognized in patients with gastrointestinal disorders. Differences may exist between patient-reported experiences and formal pharmacovigilance records. Therefore, it is of interest to evaluate self-reported ADRs among 265 adults receiving treatment for gastrointestinal conditions and compared findings with regional pharmacovigilance data from the same period. A structured and pretested survey collected demographic, clinical and ADR-related information. Overall, 51.7% reported at least one ADR, most commonly abdominal discomfort (27.2%), nausea (21.9%) and headache (17.4%). Awareness of formal ADR reporting systems was low, with only 38.5% having heard of such systems and 25.7% knowing how to report. Concordance between patient reports and pharmacovigilance data was high for gastrointestinal and central nervous system reactions but lower for severe and dermatological reactions. Female gender, higher education and poly-pharmacy were significantly associated with increased ADR reporting (p < 0.05). Thus, gaps in awareness of ADR reporting support integration of patient-reported outcomes into pharmacovigilance systems.

## Background:

Adverse drug reactions (ADRs) represent a major cause of morbidity and healthcare utilization worldwide [1]. Patients with gastrointestinal disorders are particularly vulnerable because long-term pharmacotherapy and poly-pharmacy are common in this population [2]. Proton pump inhibitors, antispasmodics, antibiotics and immunomodulatory are frequently prescribed, increasing the potential for drug-related adverse effects [3]. Pharmacovigilance systems play a critical role in monitoring drug safety after market approval [4]. These systems rely largely on voluntary reporting by healthcare professionals and patients [5]. However, underreporting remains a persistent limitation. Mild, subjective or delayed symptoms are often not documented in formal databases. As a result, the real-world burden of ADRs may be underestimated [6]. Patient-reported outcomes provide valuable insight into medication-related experiences that may not be captured in clinical trials or spontaneous reporting systems [7]. Patients may perceive symptoms differently from clinicians and may attribute common or overlapping gastrointestinal symptoms to medications [8]. Awareness of ADR reporting systems is frequently low, further contributing to gaps in pharmacovigilance data [9]. Comparing patient-reported experiences with pharmacovigilance records can identify concordance, discrepancies and potential underreporting. Such analysis may reveal patterns not evident through traditional surveillance methods. Therefore, it is of interest to compare patient-reported adverse drug reactions with pharmacovigilance records in individuals receiving treatment for gastrointestinal disorders.

## Materials and Methods:

This study uses a questionnaire to study adults with diagnosed gastrointestinal disease who were taking one or more medications prescribed to them at the time of recruitment. We approached participants at an outpatient clinic at a tertiary care facility. Participants agreed to participate and provided informed consent. We assumed a 50% prevalence of adequate awareness and perceived adverse drug reactions (ADRs). At a margin of error of 6% and a confidence level of 95%, we estimated an N of 265, while also accounting for an additional 10% for incomplete responses. We used a pre-tested structured questionnaire to collect demographics, clinical history, medications, perceived ADRs and previous reporting. The main focus of the questions was on the participants' perceptions of and experiences with drug-related symptoms. We also evaluated the questionnaire tool's consistency and clarity as part of the pre-testing process. To maintain consistency, we coded and categorized self-reported adverse drug reactions (ADRs) using standard pharmacovigilance terminology. Next, we compared patient-reported adverse drug reactions (ADRs) with entries in the National ADR Pharmacovigilance Database that were updated to the same drug classes and time periods as the patient-reported ADRs. To find trends, overlaps and differences between the survey results and the pharmacovigilance database, we entered the data into a secure database and used descriptive and inferential statistics to analyze it. The institutional ethics committee granted us ethical approval and we kept the study private at all times.

## Results:

In the study, a total of 265 participants were included, with most participants between the ages of 31 and 45 (33.6%) and with more males than females (53.6%). The most reported gastrointestinal disorders were GERD (29.4%) and IBS (23.0%) and the medications that were taken most frequently were proton pump inhibitors (68.3%). Drug reactions were reported by 137 (51.7%) of the participants, predominantly abdominal discomfort (27.2%), nausea (21.9%), headache (17.4%); less frequently fatigue (12.5%) and skin symptoms (7.2%). Awareness of pharmacovigilance reporting was low, with only 38.5% of the participants having previously heard of adverse drug reaction reporting and just 25.7% indicated they knew how to report a drug reaction, despite 70.6% agreeing that drug reactions should always be reported. Compared to records in the pharmacovigilance system, there was substantial concurrence of adverse drug reaction frequencies for proton pumps inhibitors, H2 blockers and antispasmodics, although there was more reporting of antibiotics in pharmacovigilance than was noted in our survey. The highest agreement was seen for GI-related (42.5% versus 39.8%) and CNS (23.1% versus 21.4%) reactions, while skin reactions and severe drug reactions had the lowest degree of agreement. Female participants, those with higher education and those taking three or more medications were more likely to report drug reactions, where polyp medications were the strongest association (63.4% vs. 36.6%, p=0.01). Out of the 128 participants who noted they did not report ADRs, the responses regarding why included assuming the reaction was not serious (31.7%), thinking it was unclear whether the drug was the cause of the symptom (26.0%) and not knowing how to report ADRs (21.5%). Table 1 (see PDF) show similar distributions across the age groupings and a slight male predominance. Table 2 (see PDF) shows the clinical characteristics of participants, with the most reported gastrointestinal exposures being GERD, then IBS. Table 3 (see PDF) builds off of Table 2 (see PDF) and looks at medications utilization, finding the most prescribed drug class was proton pump inhibitors. Table 4 (see PDF) highlights ADRs as self-reported by participants and just over half indicated they had an ADR. Table 5 (see PDF) identifies the types of ADRs named by participants, with abdominal discomfort, nausea and headaches being the most frequent. Table 6 (see PDF) illustrates a comparison of participant knowledge and thoughts on ADR reporting mechanisms. The data indicates that the vast majority of participants were unaware of any formal reporting mechanisms; however most participants indicated they thought ADRs should be reported by patients/ participants. Table 7 (see PDF) compares the frequency of participant reports of ADRs for comparison to entries in pharmacovigilance databases with similarities and differences for drug classes. Table 8 (see PDF) provides the comparative level of agreement between survey findings and findings from pharmacovigilance levels, with participants demonstrating a high level of agreement for ADRs involving the GI system and for the CNS, but a low level of agreement for severe and skin ADRs. Table 9 (see PDF) examines the characteristics associated with greater likelihood of reporting adverse drug reactions (ADRs). The most important variables were being female, higher education and being prescribed more medications. Finally, Table 10 (see PDF) shows the reasons participants reported that they did not report ADRs. The most common reasons reported, in terms of frequency, were they felt their symptoms were minor and they were uncertain on how to report them.

## Discussion:

This study evaluated patient-reported adverse drug reactions in gastrointestinal disorders and compared them with pharmacovigilance database records. More than half of participants reported at least one ADR. Abdominal discomfort, nausea and headache were the most frequently described symptoms. These findings reflect the high medication burden in chronic gastrointestinal disease management [10]. The overall prevalence of self-reported ADRs was 51.7%. This rate is consistent with studies indicating that chronic medication users frequently experience treatment-related symptoms. However, spontaneous pharmacovigilance systems often record lower frequencies. This difference supports the presence of underreporting in formal safety databases [11]. High concordance was observed for gastrointestinal and central nervous system reactions. This suggests that common and predictable ADRs are reliably captured in both patient reports and pharmacovigilance records. In contrast, concordance was lower for dermatological and severe reactions. Severe ADRs are rare and may require hospitalization, which influences reporting pathways. Mild dermatological reactions may be perceived as insignificant and therefore inconsistently reported [12]. A major finding was the limited awareness of ADR reporting systems. Although most participants believed ADRs should be reported, only a minority knew how to report them. This gap indicates that intention does not translate into action. Lack of knowledge regarding reporting procedures remains a critical barrier in pharmacovigilance [13]. The most common reasons for non-reporting were assuming the reaction was minor or being uncertain about causality. These findings highlight the subjective nature of symptom interpretation. Patients may normalize expected side effects or attribute symptoms to their underlying disease rather than medication. Educational reinforcement regarding ADR recognition and reporting pathways is therefore essential [14]. Demographic analysis showed that female gender, higher education level and poly-pharmacy were associated with increased ADR reporting. Women may demonstrate greater health awareness and symptom recognition. Higher education likely improves health literacy and reporting confidence. Poly-pharmacy increases exposure risk and symptom attribution probability. These findings suggest that sociodemographic factors influence pharmacovigilance engagement [15]. The comparison with pharmacovigilance data provides important context. Similar trends in drug classes such as proton pump inhibitors and antispasmodics validate patient-reported data. However, discrepancies in antibiotic-related and severe ADR categories suggest selective reporting patterns. Formal systems may preferentially capture clinically significant or medically reviewed events, whereas patient surveys capture subjective experiences [16]. Integration of patient-reported outcomes into pharmacovigilance systems may improve signal detection. Traditional spontaneous reporting relies heavily on clinician initiative. Incorporating structured patient surveys could enhance early identification of emerging safety signals. Digital health platforms and electronic reporting tools may further facilitate patient engagement [17]. This study contributes contemporary evidence by directly correlating patient-reported ADR patterns with real-world pharmacovigilance data within the same regional timeframe. Few studies have performed such parallel comparative analysis in gastrointestinal populations. The findings demonstrate that patient experience aligns with formal data for common ADRs but reveals awareness gaps and underreporting tendencies. Several limitations must be acknowledged. The study relied on self-reported data, which may introduce recall bias. Causality assessment was not formally performed using standardized algorithms. Pharmacovigilance database comparison was limited to one-year regional data. Despite these limitations, the study provides clinically relevant insights into medication safety perception. Overall, the findings emphasize that pharmacovigilance should not rely solely on spontaneous reporting systems. Patient education, simplified reporting mechanisms and integration of patient-reported outcomes are necessary to strengthen drug safety monitoring. Addressing awareness deficits may improve reporting behavior and enhance public health surveillance.

## Conclusion:

Self-reported adverse drug reactions among patients with gastrointestinal disorders show substantial overlap with pharmacovigilance records, particularly for common gastrointestinal and central nervous system symptoms. However, awareness of formal ADR reporting systems remains low and underreporting persists despite recognition of the importance of reporting. Female gender, higher education and poly- pharmacy are associated with increased likelihood of ADR reporting. Integrating patient-reported outcomes into pharmacovigilance systems and strengthening education on reporting pathways may enhance signal detection and improve medication safety surveillance.
